# Flux balance analysis of primary metabolism in *Chlamydomonas reinhardtii*

**DOI:** 10.1186/1752-0509-3-4

**Published:** 2009-01-07

**Authors:** Nanette R Boyle, John A Morgan

**Affiliations:** 1 School of Chemical Engineering, Purdue University, 480 Stadium Mall Drive, West Lafayette, Indiana 47907, USA

## Abstract

**Background:**

Photosynthetic organisms convert atmospheric carbon dioxide into numerous metabolites along the pathways to make new biomass. Aquatic photosynthetic organisms, which fix almost half of global inorganic carbon, have great potential: as a carbon dioxide fixation method, for the economical production of chemicals, or as a source for lipids and starch which can then be converted to biofuels. To harness this potential through metabolic engineering and to maximize production, a more thorough understanding of photosynthetic metabolism must first be achieved. A model algal species, *C. reinhardtii*, was chosen and the metabolic network reconstructed. Intracellular fluxes were then calculated using flux balance analysis (FBA).

**Results:**

The metabolic network of primary metabolism for a green alga, *C. reinhardtii*, was reconstructed using genomic and biochemical information. The reconstructed network accounts for the intracellular localization of enzymes to three compartments and includes 484 metabolic reactions and 458 intracellular metabolites. Based on BLAST searches, one newly annotated enzyme (fructose-1,6-bisphosphatase) was added to the *Chlamydomonas reinhardtii *database. FBA was used to predict metabolic fluxes under three growth conditions, autotrophic, heterotrophic and mixotrophic growth. Biomass yields ranged from 28.9 g per mole C for autotrophic growth to 15 g per mole C for heterotrophic growth.

**Conclusion:**

The flux balance analysis model of central and intermediary metabolism in *C. reinhardtii *is the first such model for algae and the first model to include three metabolically active compartments. In addition to providing estimates of intracellular fluxes, metabolic reconstruction and modelling efforts also provide a comprehensive method for annotation of genome databases. As a result of our reconstruction, one new enzyme was annotated in the database and several others were found to be missing; implying new pathways or non-conserved enzymes. The use of FBA to estimate intracellular fluxes also provides flux values that can be used as a starting point for rational engineering of *C. reinhardtii*. From these initial estimates, it is clear that aerobic heterotrophic growth on acetate has a low yield on carbon, while mixotrophically and autotrophically grown cells are significantly more carbon efficient.

## Background

Algae and other marine organisms are responsible for the fixation of almost half of the inorganic carbon from the atmosphere [1]. With rising atmospheric carbon dioxide levels, knowledge of how photosynthetic organisms convert atmospheric carbon dioxide into metabolites and other important compounds is becoming increasingly important. Not only do these organisms fix carbon dioxide, but they also have the potential to be used for the production of inexpensive bulk chemicals because the major inputs into the system (light and CO_2_) are essentially free. However, to harness this potential through metabolic engineering, a deeper understanding of photosynthetic metabolism is required.

There are several widely accepted methods for modelling metabolism, ranging from highly detailed kinetic models to less complex stoichiometric models. One of the more increasingly used methods is flux balance analysis (FBA), which has the ability to predict fluxes using linear programming with the knowledge of reaction stoichiometry, biomass composition and additional constraints, such as limits on uptake/excretion rates and thermodynamic constraints. FBA has been used for a number of model organisms [2-7] to predict fluxes and viability of knockouts. FBA can also be used for rational strain design, both to predict theoretical yields and to identify bottlenecks or sinks in metabolism that need to be altered to achieve the theoretical yield [8,9].

FBA has been previously used to model photosynthetic metabolism in a model cyanobacteria, *Synechocystis *[9]. In an earlier related study, the metabolic network of another cyanobacterium, *Arthrospira platensis*, was reconstructed and fluxes computed [10]. The goal of the current study was not only to model photosynthetic metabolism, but to model it in a higher eukaryote in order to have a model more representative of plant metabolism. Therefore, *Chlamydomonas reinhardtii *was chosen as a representative algal species for this study. *C. reinhardtii *has been used as a model organism to study numerous cellular functions from photosynthesis research to flagellar function and assembly [11] and most recently a metabolomics and proteomics approach to genome annotation [12]. It has served as a bridge between higher plants and cyanobacteria in the field of photosynthetic research due to the relative simplicity of the cell structure and metabolism while being more comparable to higher plants. *C. reinhardtii *was the first algal species to have its genome sequenced [13] and this has provided researchers with an abundance of data on genes and their functions. Another advantage of *C. reinhardtii *is that its photosynthetic capability is dispensable; as it can grow heterotrophically on acetate. However, as an acetate flagellate, it can only grow on acetate and similar 2-carbon molecules in the dark. In the presence of light, *C. reinhardtii *can metabolize pentoses and hexoses (mixotrophic growth) as well as acetate [14] and supports autotrophic growth using carbon dioxide as the carbon source.

The major contribution of this work is the reconstruction of a compartmental metabolic network for primary metabolism in the green alga, *C. reinhardtii*. The metabolic network was reconstructed using the genomic database [13], biochemical texts [15-17], metabolic pathway databases [18,19], and archival journal articles (See methods section for specific articles). Localization of enzymes in the cell was proposed using bioinformatic software [20,21]. FBA was then used to predict flux distributions for three conditions: autotrophic, heterotrophic and mixotrophic growth.

## Results and discussion

### Network reconstruction

**Figure 1:**
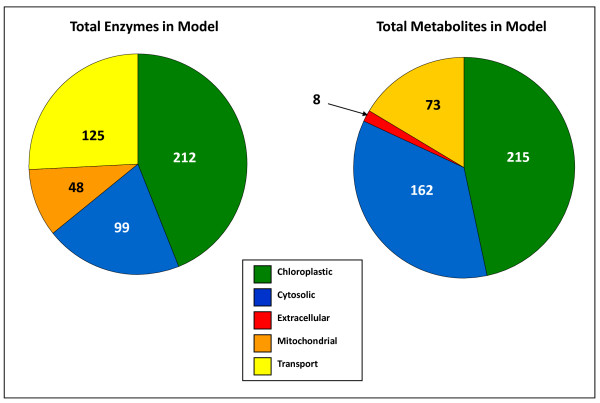
**Distribution of enzymes and metabolites**. Distribution of enzymes and metabolites in the reconstructed model of *Chlamydomonas reinhardtii*. Almost half of both the enzymes and metabolites are localized to the chloroplast, followed by the cytosol and mitochondria. There are also a large number of transport reactions, indicating the importance of metabolite exchange between compartments.

The reconstructed metabolic network of *C. reinhardtii *consists of 458 metabolites and 484 metabolic reactions. Almost half of the metabolites included in the network are present in the chloroplast (Figure 1), which is a result of the large number of reactions localized to the chloroplast (212 out of 484). The cytosol acts as the 'hub' of transport for metabolites as well as the polymerization location for most macromolecules; as a result, roughly one third of the metabolites in the model are localized there. Another significant portion of reactions in the model function as intracellular transporters, which indicates the high interconnectivity between the compartments.

In the course of reconstructing the model, several assumptions about the presence or absence of reactions had to be made. Although many reactions were not linked to the EC number in the database, the coinciding gene was determined by performing a protein BLAST [22] search. Of the 359 metabolic reactions in the model, only 17 resulted in no hits in the database, these are shown in Table 1. One enzyme, fructose-1,6-bisphosphatase resulted in a hit in the database but was not previously annotated and was subsequently added to the *C. reinhardtii *database. Finally, a few reactions/enzymes/metabolites were assumed to be present as formulated in the model, which include the electron transport chain (ETC) reactions, oxidative phosphorylation and a simplified lipid biosynthesis reaction.

**Table 1:** Missing enzymes from the *C. reinhardtii *database

**Rxn #**	**E.C. #**	**Gene Description**	**Reaction**
5	2.7.1.11	Phosphofructokinase	F6P + ATP --> F16P + ADP

6	3.1.3.11	Fructose-1,6-Phosphatase	F16P + H2O --> F6P + Pi

7	2.7.1.11	Phosphofructosekinase	F6P_c + ATP_c --> F16P_c + ADP_c

167	3.6.1.3	ATPase	ATP_c + H2O_c --> ADP_c + Pi_c

169	3.6.1.3	ATPase	ATP_m+ H2O_m --> ADP_m + Pi_m

209	2.7.1.31	Glycerate Kinase	Glycerate_c + ATP_c → 3PG_c + ADP_c

300	4.1.2.15	DAP synthase, KDPH Synthetase	PEP_c + E4P_c + H2O_c --> DDP_c

326	3.6.1.-	Dihydroneopterin Dephosphorylase	DHN + 2 H2O --> DHDN + PP + Pi

328	2.7.6.3	Dihydropterin Pyrophosphokinase	HMD + ATP --> DHT + AMP

330	4.1.3.38	Aminodeoxychorismate Lyase	4AD --> PYR + ABZ

336	2.7.1.28	Triose Kinase	Glyceraldehyde + ATP --> GAP + ADP

353	2.3.1.31	Homoserine O-acetyltransferase	Hser + AcCoA --> OAH + CoA

357	2.3.1.46	Homoserine O-Succinyltransferase	SucCoA_c + Hser_c --> CoA_c + OSH_c

377	4.1.1.48	Indole-3-glycerol Phosphate Synthase	CPDRP_c --> I3GP_c + H2O_c + CO2_c

380	3.1.3.15	Histidinol Phosphatase	HOLP_c + H2O_c --> HOL_c + Pi_c

446	2.7.4.14	Cytidylate kinase.	CMP_c + ATP_c --> CDP_c + ADP_c

484	6.3.4.3	Formate-tetrahydrofolate ligase	ATP_c + Formate_c + THF_c --> ADP_c + Pi_c + FTHF_c

Missing enzymes from the *Chamydomonas reinhardtii *database but assumed present to make a complete metabolic network. Metabolites without subscripts are localized in the cytosol, those denoted by a small 'm' and 'c' are localized to the mitochondria and chloroplast.

### Localization of enzymes and metabolites

**Figure 2:**
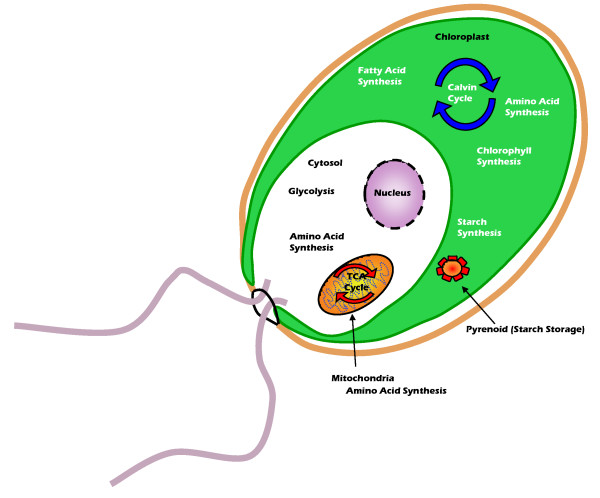
**Reconstructed metabolic network of *C. reinhardtii***. Based on predicted target peptide sequences, the following localization of pathways was determined. Chloroplast: fatty acid synthesis, amino acid synthesis, nucleotide synthesis, starch synthesis and chlorophyll synthesis. Mitochondria: TCA cycle, amino acid synthesis. Cytosol: glycolysis, amino acid synthesis and fatty acid synthesis.

The reactions in the network were localized into three compartments; cytosol, mitochondria and chloroplast (see Figure 2). Localization of enzymes present in the database was determined by submitting the amino acid sequence to software programs [20,21] which identify the presence or absence of a signal peptide (SP). Enzymes that did not have a predicted SP or those that were targeted to the secretory pathway were modelled as cytosolic because only three compartments were considered. Predicting the localization of an enzyme is only possible when the amino acid sequence is known, therefore for the few enzymes that were not in the database, the localization was assumed based on where the preceding and following metabolic steps were located. Metabolites were also assigned to compartments based on which compartment they were in while participating in enzymatic reactions; these are differentiated by a small m (mitochondria) or c (chloroplast) following the metabolite abbreviation. Metabolites without a subscript, by default, are located in the cytosol [see additional file 1 for a complete list of metabolites]. Metabolites were allowed to move between compartments either through known transporters and shuttle systems or through inferred reactions (or passive diffusion) based on the need for metabolites in certain compartments.

### Biomass formation equation

The macromolecular composition of cells from each growth condition was measured as described in the methods section. The DNA and RNA content were assumed constant across all growth conditions. The final cellular dry weight composition of each growth condition is given in Table 2. The elemental composition was also measured (Table 3) and compared to the values calculated from the measured cellular composition. The final balances varied from the measured elemental composition by 0.1% to 6%. The biomass composition was then used to construct a biomass formation equation for each growth condition. Along with the 7 main components (DNA, RNA, protein, lipid, chlorophyll a, and chlorophyll b), the polymerization and growth associated maintenance energy was also included in the biomass formation equation. The polymerization energy requirement for protein, DNA and RNA from their respective precursors was assumed to be the same as that for *E. coli *[23]. The resulting biomass formation equations for auto-, hetero- and mixotrophic growth are shown in Table 4.

**Table 2:** Dry weight composition

	**Fraction DW**					
	**Autotrophic**		**Mixotrophic**		**Heterotrophic**	

**Cell Component**	**Average**	**(+/-)**	**Average**	**(+/-)**	**Average**	**(+/-)**

Carbohydrate	0.508		0.381		0.448	

Protein	0.261	0.014	0.303	0.027	0.222	0.007

Lipid	0.189	0.016	0.279	0.023	0.287	0.018

Chlorophyll a	0.009	5.6E-06	0.007	4.2E-05	0.018	3.7E-04

Chlorophyll b	0.015	2.5E-04	0.013	4.7E-05	0.008	3.6E-04

Measured and calculated dry weight composition based on mass fraction from three independent measurements +/- one standard deviation.

**Table 3:** Elemental composition

	**C**	**H**	**N**	**O**
**Autotrophic**	0.481	0.073	0.058	0.388

**Mixotrophic**	0.507	0.079	0.035	0.379

**Heterotrophic**	0.505	0.077	0.105	0.313

Elemental composition of different growth regimes. Carbon, hydrogen and nitrogen were measured directly, oxygen was taken to be the balance.

**Table 4:** Biomass formation equations

	**Biomass Formation Equation (moles/kg biomass)**		
	
	**Autotrophic**	**Mixotrophic**	**Heterotrophic**
DNA	0.002	0.002	0.002

RNA	0.051	0.051	0.051

Protein	2.005	2.328	1.706

Carbohydrate	2.008	1.513	1.752

Lipid	0.203	0.298	0.307

Chlorophyll a	0.010	0.008	0.020

Chlorophyll b	0.016	0.014	0.009

ATP (polymerization)	9.350	13.320	8.890

ATP (maintenance)	29.890	29.890	29.890

Biomass formation equation given in moles per kg biomass. Protein, lipid, chlorophyll were measured independently for each growth regime. DNA and RNA content were assumed the same for all cases and carbohydrates was assumed to be the balance. Polymerization energy includes energy required for protein, RNA and DNA polymerization and maintenance is based on a fitted value for heterotrophic growth.

### Simulation results

#### Central metabolism flux maps

Flux maps for three growth conditions (auto-, hetero-, and mixotrophic) were calculated using the reconstructed network and FBA. During autotrophic growth, the cell fixes carbon dioxide by converting light into cellular energy (reducing equivalents and ATP). In this study we defined heterotrophic growth as aerobic growth on acetate in the dark; the cell using acetate for both carbon and energy sources. Another metabolic mode, mixotrophic growth, is the link between the two extremes. In our model, mixotrophic growth has three inputs: light, acetate and carbon dioxide.

**Figure 3:**
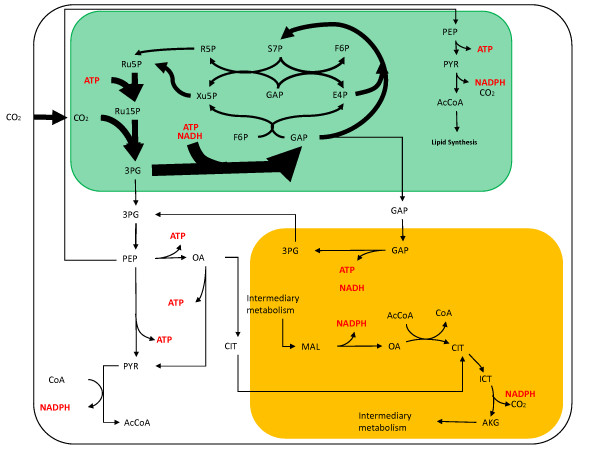
**Autotrophic central metabolism flux map**. The thickness of the arrows has been normalized to the total carbon dioxide uptake of 100 moles. The green compartment represents the chloroplast and the orange compartment is the mitochondria.

Autotrophic growth was simulated using a two-step optimization with a basis of 100 moles CO_2_. Interestingly, the fluxes for both optimization steps were identical (as reported previously by [9]), which implies the cell is optimally utilizing energy to produce biomass without needing the second constraint of minimum light energy. As expected, the majority of the carbon flux is directed through the Calvin Cycle (Figure 3). The energy required for the regeneration of GAP from 3PG to run the Calvin Cycle is supplied by photophosphorylation. Due to compartmentation and no known direct NADPH or NADH transporters, the flux through the non-cyclic ETC is almost wholly constrained by the flux from 3PG to GAP, which is the main consumption of NADH in the chloroplast. The rest of the NADPH produced by the non-cyclic ETC must be transported out of the chloroplast via an indirect shuttle. In the autotrophic case, the cell produces most of its energy from the conversion of light energy, which occurs in the chloroplast. However, there is demand for both ATP and NAD(P)H outside the chloroplast for other biosynthetic reactions. Indirect transport of this energy is accomplished by transporting GAP from the chloroplast to the mitochondria and subsequently degrading it to 3PG, releasing both ATP and NADH into the mitochondria. The calculated photosynthetic quotient (moles of oxygen released per mole carbon dioxide fixed) for the optimal flux distribution is 1.27 which agrees with the typical range of 1.0 – 1.8 for algae [24].

**Figure 4:**
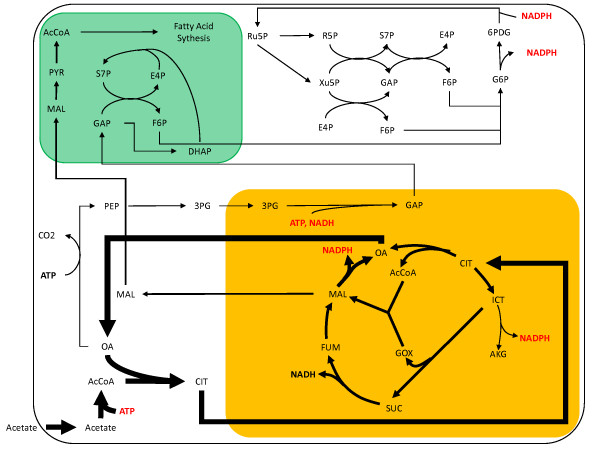
**Heterotrophic central metabolism flux map**. The thickness of the arrows has been normalized to the total acetate uptake of 100 moles. The green compartment represents the chloroplast and the orange compartment is the mitochondria.

The basis of the heterotrophic simulation was 100 moles acetate because *C. reinhardtii *is only capable of growing heterotrophically on acetate and other similar 2-carbon molecules. Most of the carbon flux for heterotrophic growth is directed through the TCA cycle, as would be expected (Figure 4). Since the cell is not capable of metabolizing external sugars in the dark, almost all the energy is produced by respiration in the TCA cycle. The oxidative pentose phosphate pathway is also active, providing reducing power for use in the cytosol. Synthesis of G6P occurs via gluconeogenesis; a lack of ATP and NADH in the cytosol causes the regeneration of GAP from 3PG to take place in the mitochondria. The glyoxylate shunt is also active, which is known to be needed to metabolize acetate in *E. coli *[25], *Neurospora crassa *[26],*Scenedesmus obliquus *[27], *A. thaliana *[28] and several other organisms.

**Figure 5:**
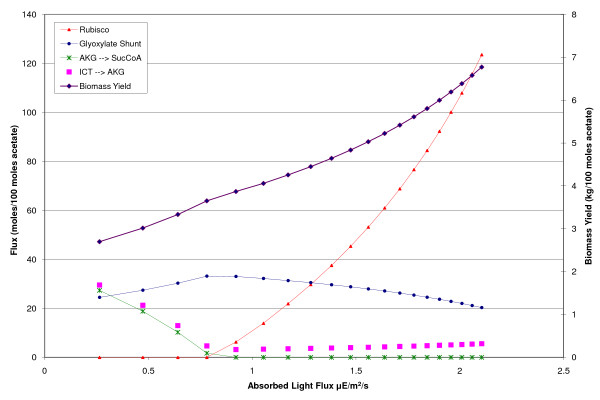
**Mixotrophic growth as a function of absorbed light**. Mixotrophic growth in *C. reinhardtii *has two distinct regions. The first region (below 0.8 μE/m2/s) is characterized by a complete TCA cycle and inactive Rubsico. The second region (above 0.8 μE/m2/s) has an incomplete TCA cycle due to the zero flux through oxoglutarate decarboxylase and an active Rubisco.

*C. reinhardtii *is also capable of mixotrophic growth, utilizing acetate, light and carbon dioxide for growth. Mixotrophic growth was simulated by using heterotrophic growth as the base case and allowing the uptake of carbon dioxide and light for energy. The free uptake of carbon dioxide was allowed, however to limit the biomass formed, an additional constraint on the absorbed light was added. The total absorbed light flux was fixed over a range of 0 to 2 μE/m^2^/s in a step-wise fashion and at each light flux, the optimal flux distribution was calculated. Fluxes that changed significantly over the range of absorbed light are plotted in Figure 5. From this graph it is evident there are two distinct regions of growth. The first resembles heterotrophic growth in which carbon fixation does not occur and the cell is producing CO_2_. However, unlike the heterotrophic case above, at very low light levels, the cell has a complete TCA cycle. The flux through 2-oxoglutarate decarboxylase decreases steadily to zero at which point the flux begins to be directed through Rubisco. This could be due to the need for NAD(P)H for biomass synthesis during low light conditions, but when light intensity increases enough to send flux through Rubisco, the cell is capable of producing enough NADPH through the non-cyclic ETC to supply metabolism with NADH via transhydrogenases. In the heterotrophic case, *C. reinhardtii *has an incomplete TCA cycle. This could also be a result of the production of NADPH within the chloroplast and the subsequent indirect transport of reducing equivalents throughout the cell. At a light flux of approximately 0.8 μE/m^2^/s, flux is directed through the Calvin cycle and the cell enters the second growth regime. In this growth regime, the glyoxylate shunt flux steadily decreases while the Rubisco flux increases rapidly with increasing light. As the light increases, the flux distribution becomes more similar to the autotrophic case. The increase in biomass flux with each increase in light is slightly less than that for the first growth regime. This is due to the higher energetic demand of carbon fixation.

Quantitative results for all three growth regimes as well as reaction lists can be found at .

#### Comparison of yields

The autotrophic biomass yield is 28.9 g biomass for every mole of carbon taken up (Table 5), based on the elemental analysis of *C. reinhardtii*, this is 100% of the carbon taken into the cell. This is of course due to the production of energy from light during photosynthesis, so no net carbon is lost during respiration. In contrast, the heterotrophic biomass yield is 15 g per mole carbon, which implies that almost half of the carbon taken in by the cell is used for energy production instead of biomass formation. The fraction of carbon used for energy production is quite high compared to another photosynthetic organism, Synechocystis [9], which only utilized 37% of the carbon for energy. This is due to the difference in energy content of the substrate. *Synechocystis *utilizes glucose, which has significantly higher energy content per mole than acetate; glucose has a standard heat of combustion of -2.8 kJ/mole compared to -0.8 kJ/mole for acetate. During mixotrophic growth, the biomass yield of *C. reinhardtii *increases from 13.5 to 22.9 g per mole carbon. With increasing light flux, the cell can direct more carbon towards biomass and less towards energy production, however, the amount of carbon fixed per photon is constant. Therefore, the maximum yield is lower than the autotrophic yield because it is limited by the energy in the cell. During mixotrophic growth, the cell must utilize acetate and it has to divert some carbon away from biomass and towards energy production.

**Table 5:** Biomass yields

**Growth Condition**	**Yield (g biomass/mole carbon)**
Autotrophic	28.9

Heterotrophic	15.6

Mixotrophic	Increases with increasing light flux from 13.5 to a maximum of 22.9

Biomass yields under different growth conditions. The organic carbon source for heterotrophic and mixotrophic growth was exogenously supplied acetate.

#### Comparison to a model photosynthetic microbe

Flux estimates for autotrophic growth were compared to estimated fluxes for the cyanobacteria, Synechocystis sp PCC 6803 [9]. One major difference is the flux through the cyclic and non-cyclic ETCs (Table 6). Although both organisms utilize approximately the same amount of energy to produce each kilogram of biomass, the flux through the ETCs are split quite differently. *Synechocystis *has a much higher flux through the non-cyclic ETC than *C. reinhardtii *which is due mainly to the compartmentation in the model; due to a lack of a direct NAD(P)H transporter in *C. reinhardtii*, the flux through the non-cyclic ETC is constrained to match the need for NADPH in the chloroplast. Any additional NAD(P)H is indirectly transported via shuttles, but these shuttles are also constrained by mass balances and steady state assumptions. In contrast, since Synechocystis is prokaryotic and unicellular, it can use the non-cyclic ETC to produce all the NADPH needed in the cell. Therefore, to make up for the energetic difference of having a lower flux through the non-cyclic ETC, *C. reinhardtii *must have a larger flux through the cyclic ETC. Cellular compartmentation also comes into play in the total moles of oxygen produced. The only reaction the cell can use to produce oxygen in both organisms is the non-cyclic ETC, which is why the production of oxygen from *C. reinhardtii *is lower than that of Synechocystis. Comparison of the biomass yields per 100 moles carbon dioxide from both organisms shows yet another difference; Synechocystis has a lower yield than *C. reinhardtii*, 2.43 kg and 2.89 kg respectively. Part of this difference can be explained by the use of a lumped biomass equation for Synechocystis which specifies the loss of approximately one mole of carbon dioxide per kilogram biomass formed in order to have a balanced reaction. In contrast, the *C. reinhardtii *model is much more detailed and the carbon dioxide lost during biosynthesis can be fixed because it is not required to be present on the right hand side of the biomass formation equation to balance the reaction. This results in 2.47 moles of carbon dioxide loss for the production of 2.43 kilograms of *Synechocystis*, which translates to a loss of 0.11 kg of biomass. Another element that contributes to the difference in yield is the carbon content of the 2 organisms; Synechocystis is reported to be 51% carbon [9] while *C. reinhardtii *was measured to be 48% carbon, which explains at least 0.15 kilograms difference in biomass yield. Due to the nature of the optimization technique employed, which allows the cell to use unlimited energy in the first step, the difference in yields can be attributed to these two factors and it is not due to a lack of energy.

**Table 6:** Comparison of selected fluxes to *Synechocystis*

	**Autotrophic growth fluxes (moles/100 moles CO_2_/kg biomass)**	
	
	***Synechocystis***	***C. reinhardtii***
**Cyclic ETC (photons)**	54	192

**Non-cyclic ETC (photons)**	480	352

**Total photons**	534	544

**O_2 _released**	60	44

Comparison of fluxes during autotrophic growth for *Synechocystis *and *C. reinhardtii*. The fluxes are normalized per kilogram biomass produced.

## Conclusion

A stoichiometric model of primary metabolism was constructed for *C. reinhardtii *from the genomic database, pathway databases and literature. The network includes all the major pathways in central metabolism (glycolysis, TCA cycle, oxidative and reductive pentose phosphate pathways) as well as amino acid, nucleotide, chlorophyll, lipid and starch synthesis. Metabolic network reconstruction is a valuable tool to identify gaps in existing knowledge [4,5,29-31]. As a result of the reconstruction process, one new gene was annotated and 16 other genes were identified to be missing, implying either non-conserved amino acids sequences or possibly new pathways. Despite being incomplete, it is the first model of a eukaryotic photosynthetic organism to include central and intermediary metabolism with three metabolically active compartments.

Intracellular fluxes were estimated using FBA for three growth conditions, autotrophic, heterotrophic, and mixotrophic. Yield on carbon and growth rate are factors that need to be considered in choosing the appropriate growth conditions for maximizing the production of desired metabolites. For example, a lower yield for heterotrophic growth is off-set by a faster growth rate, which may be ideal for the production of growth associated products. Along with this, the model provides a more complete picture of photosynthesis in a compartmented organism and can serve as a starting point for models of other photosynthetic algae and more complex models of higher photosynthetic organisms.

With renewed interest in biofuel production from algae [32] the reconstructed network of *C. reinhardtii *presented here can serve as a starting point for metabolic engineering of lipid or starch production in algae. Future work will use elementary mode analysis [33] to determine if multiple pathways that lead to the same optimum exist, which is highly likely due to the size and complexity of the network.

## Methods

### Network reconstruction

A stoichiometric model of the primary metabolism of *C. reinhardtii *was constructed using the genomic database [13], pathway databases [18,34], biochemistry texts [15-17,35] and archival journal articles. The reconstruction process began with a search of the genome database for reactions in the metabolic pathways to be modelled. This included the following pathways: glycolysis, gluconeogenesis, pentose phosphate pathway (oxidative and reductive), TCA cycle, photorespiration, glycolate cycle (recycles 2-phosphoglycolate to 3-phosphoglycerate in plants) and the biosynthesis of amino acids, chlorophyll, nucleotides, starch and lipids. The reversibility of reactions was also assigned during this initial search; if no information was available, reactions were assumed to be reversible. Starch and lipid metabolism were simplified by making a few assumptions. An average chain length of 50 was assumed for starch based on typical values that range from 3–1000 for amylase chains and 3–50 for amylopectin [36]. Fatty acid synthesis reactions were added to represent the synthesis of hexadecanoic and octadecanoic acids as well as their corresponding unsaturated fatty acids (16:1, 16:2, 16:3, 16:4, 18:1, 18:2, 18:3, 18:4), which represent the majority of fatty acids present in *Chlamydomonas reinhardtii *[37]. The synthesis of the major classes of lipids (MGDG, DGDG, SQDG, PG, PI, DGTS, and PE) were included based on average lipid composition for each head group and the localization of each lipid was based on the distribution of C_16 _and C_18 _in the C1 and C2 position on the head group [37]. Although detailed lipid synthesis reactions were included in the network reconstruction, lipids were lumped into a single representative lipid to simplify the FBA simulations. An 'average' lipid made up of unsaturated C18 fatty acids and a glycerol head group was assumed based on the largest percentage of lipids being C18 as reported by Janero and Barrnett [38] and is shown in reaction 179 in additional file 2.

Despite large efforts to fully annotate genome databases, not all enzymatic functions are listed and therefore gaps exist in the pathways. Gaps in the network were first addressed by searching pathway databases for the missing enzymes and corresponding genes in other organisms whose genome is sequenced (*A. thaliana, E. coli, and S. cerevisiae*). The amino acid sequence of these known genes were then blasted against the *C. reinhardtii *database; in most cases, this resulted in a hit which had already been annotated but not linked to the KEGG portion of the genome database. There were a few genes that resulted in hits to proteins that were either listed as having a different function or were not annotated at all (see results). A few enzymes resulted in no hits in the database, but were assumed to be present in order to have a complete network.

### Phosphorylation

Accurate reconstruction of a metabolic network requires the inclusion of reactions for cellular energy production. Two main sources of energy in algae are photophosphorylation and oxidative phosphorylation. Photophosphorylation is the process by which light is converted into energy (ATP) and reducing power (NADPH) via two electron transport chains (ETC). The cyclic ETC is made up of several membrane-bound and membrane associated proteins that are coupled with the light harvesting complex, photosystem I (PSI) which pumps protons across the chloroplast membrane. The non-cyclic ETC uses both light harvesting complexes (photosystem I and photosystem II) to produce NADPH and pump protons across the plastidic membrane. This process has been modelled previously in *Synechocystis *by Shastri and Morgan (2005) as two non-interacting reactions as shown below:

(1) $$1\text{ absorbed photon}\to {\text{2 H}}_{\text{c}}^{+}$$

(2) $$4\text{ absorbed photons}+{\text{NADP}}_{\text{c}}^{+}+{\text{H}}_{\text{2}}{\text{O}}_{\text{c}}\to {\text{NADPH}}_{c}+{\text{6 H}}_{\text{c}}^{+}+\text{0}{\text{.5 O}}_{2c}$$

Both the cyclic and non-cyclic ETCs are coupled to the chloroplast ATP synthase complex to synthesize ATP from ADP. This enzyme complex couples the translocation of protons with the production of ATP. Recent studies have shown that the H^+^/ATP ratio for this reaction is 14/3 or 4.67 [39]. The ATP synthase reaction can then be modelled as:

(3) $$4.67{\text{ H}}_{\text{c}}^{+}+{\text{Pi}}_{\text{c}}+{\text{ADP}}_{\text{c}}\to {\text{ATP}}_{\text{c}}$$

Oxidative phosphorylation occurs via the mitochondrial ETC, which is made up of 4 complexes (I, II, III, and IV) that control the flow of electrons from NADH to reduce oxygen to water. The ETC is also coupled to an ATP synthase which produces ATP from ADP by pumping protons across the mitochondria membrane. Although the exact H^+^/ATP ratio for the mitochondria ATP synthase has not yet been agreed upon, it is assumed to be between 3 and 4 [39-41]. For this model, the H^+^/ATP ratio of the reaction was assumed to be the same as that used in the genome-scale *Saccharomyces cerevisiae *model [41]. The reaction in the model is shown in equation 4.

(4) $${\text{3 H}}_{\text{m}}^{+}+{\text{Pi}}_{\text{m}}+{\text{ADP}}_{\text{m}}\to {\text{ATP}}_{\text{m}}$$

#### Linear programming formulation

The reconstructed metabolic network provided the information necessary to develop a stoichiometric model [42]. A stoichiometric model takes the form S·v = 0, where S is the stoichiometric matrix and v is a vector of fluxes. The stoichiometric matrix and flux vector is constructed by writing a steady-state mass balance on each intracellular metabolite in each compartment, this set of mass balances is then converted to matrix form. Reversible reactions are separated into one forward and one reverse reaction in order to constrain all fluxes to be positive, therefore minimizing solution time. An additional constraint was added to only allow one direction of a reversible reaction to be active by introducing a binary variable [9]. Since the resulting model was underdetermined, linear programming was used to solve for optimal fluxes.

$$\begin{array}{l} \text{Maximize biomass subject to:}\\ \sum_{j}{\text{s}}_{\text{ij}}\upsilon_{j}=0\text{ for every i}\in {\text{M}}_{\text{i}}\\ \sum_{j}{\text{s}}_{\text{ij}}\upsilon_{j}\leq 0\text{ for every i}\in {\text{M}}_{\text{r}}\\ \sum_{j}{\text{s}}_{\text{ij}}\upsilon_{j}\geq 0\text{ for every i}\in {\text{M}}_{\text{p}}\\ \upsilon_{j}\geq 0 \end{array}$$

where s_ij _is the stoichiometric coefficient of the i^th ^metabolite in the j^th ^reaction, v_j _is the flux of the j^th ^reaction, M_i _is the set of intracellular metabolites, M_r _is the set of reactants other than substrate, and M_p _is the set of products excreted.

A mixed integer linear program was formulated in the GAMS environment (GAMS Development Corporation, Washington, DC) and the optimum solution was found using the ILOG CPLEX 8.100 solver (ILOG, Inc. Mountain View, CA).

#### Optimization

Unlike heterotrophic organisms that utilize the same substrate as the source of both carbon and energy, photoautotrophic organisms require two substrates, one for energy (light) and one for carbon (carbon dioxide). Due to the input of two substrates, FBA simulations can be run in either light or carbon limitation conditions. To simulate carbon limitation, the model is allowed unlimited light, which calculates an optimal biomass flux. No fermentation products were detected in the media during growth (see maximum uptake rates discussion) therefore, in the absence of carbon overflow products, the yield of biomass is fixed because the only outlet for carbon is biomass. For photoautotrophic metabolism, a more meaningful result is to find the flux distribution that maximizes biomass while minimizing energy usage. Therefore the optimization is done in two steps. The first step is to maximize biomass with no constraint on light and the second is to fix the biomass and minimize light. Flux distributions for the heterotrophic case are the result of a one-step optimization to maximize biomass.

#### Culture conditions

*Chlamydomonas reinhardtii *strain CC-400 cw15 mt+ was acquired from the *Chlamydomonas *Genetics Center. Cells were cultivated at 25°C in 250 ml flasks with a working volume of 50 ml and an agitation rate of 200 RPM. Heterotrophic and mixotrophic cells were grown in TAP media [43] and autotrophic cells were grown in similar media without addition of acetic acid. Mixotrophic and autotrophic cultures were grown under constant illumination at an average fluence rate of 65 μE/m^2^/s. All cells were grown in the presence of atmospheric carbon dioxide levels. Growth was monitored spectrophotometrically by measuring absorbance at 750 nm.

#### Maximum uptake rates

In order to add constraints on nutrient and light uptake to the model, additional experimental measurements were taken. Maximum growth rates were measured for all three growth conditions from three separate experiments. The results are shown in Table 7. For autotrophic growth, the maximum carbon dioxide uptake rate was calculated to be 2.04 mmol/g biomass/hr. The maximum solar light flux was set to be 2100 μE/m^2^/s [44]. For heterotrophic growth, the maximum acetate uptake rate was measured using high performance liquid chromatography (HPLC) coupled to an refractive index detector (RID) detector and was found to be 12.06 mmol/g biomass/hr. The same HPLC method was used to look for fermentation products, but none were detected and therefore they were not included in the model. The biomass growth yield on acetate was measured in exponentially growing cells to be 3.12 ± 0.23 kg biomass/100 moles acetate from three independent experiments.

**Table 7:** Specific growth rates

**Growth Regime**	**Growth Rate (hr^-1^)**
Heterotrophic	0.035 ± 0.002

Autotrophic	0.059 ± 0.001

Mixotrophic	0.066 ± 0.007

Experimentally determined specific growth rates for different cultivation conditions.

#### Estimation of cell surface area

In order to convert the calculated total photons from the model to a flux (μE/m^2^/s), the surface area per kilogram biomass must be calculated. Based on experimental measurements, the dry weight of a typical *C. reinhardtii *was determined to be 0.2 pg. The length and width of cell were assumed to be 10 μm and 3 μm respectively [43] based on literature values. The geometry of the cell was assumed to be a prolate spheroid. The surface area per kilogram biomass was then calculated to be 389 m^2^kg^-1^.

#### Maintenance requirements

Growth associated and non-growth associated maintenance requirements were also included in the model. Growth associated energy is included to account for partially unknown energy requirements for transport, biosynthesis and polymerization [42] while non-growth associated accounts for cellular maintenance operations such as DNA repair, cell wall maintenance, and pH control. Growth associated maintenance was found to be 29.89 mmol ATP/g biomass by fitting the model to the experimentally determined biomass yield by changing the ATP requirement. This value falls into the range of published values for growth associated maintenance values [2-7]. Autotrophic and mixotrophic maintenance requirements were assumed to be the same. Non-growth associated maintenance requirements range from 0.36 mmol ATP/g DW hr for *Lactobacillus plantarum *to 7.60 mmol ATP/g DW for *E. coli*. For *C. reinhardtii*, non-growth associated maintenance was assumed to be 1.50 mmol ATP/g DW [3,5,7].

#### Biomass composition

The biomass composition was determined separately for each of the three growth regimes: autotrophic, mixotrophic and heterotrophic growth. Lipids were measured using the chloroform-methanol extraction method of Ishida *et al*. [45]. The resulting water layer and pellet were then dried and resuspended in 0.2 N NaOH and diluted by a factor of 5. This solution was then assayed for protein content with the Pierce BCA protein assay kit (Pierce Biotechnology, Inc. Rockford, IL). The amino acid composition (Additional file 3) was estimated from Gas chromatography-mass spectrometry (GC-MS) analysis of hydrolyzed protein (data not shown). Chlorophyll a and b were measured [43] and subtracted from the total lipid measurement. DNA and RNA were assumed to be constant for all growth conditions; the DNA content was determined by Chiang et al[46] to be 1.23 × 10^-7 ^μg per cell and the RNA content was assumed to be 28-fold higher than the DNA content [47]. The GC content of DNA was measured to be 62.1% [43] and the same GC content was assumed for RNA. Carbohydrate composition was calculated as the balance of the fraction dry weight. The elemental composition of lyophilized cells was also determined. In all cases except elemental composition, experiments were done in triplicate.

## Abbreviations

3PG: 3-Phosphoglycerate; ATP: Adenosine Triphosphate; BLAST: Basic Local Alignment Search Tool; CO_2_: Carbon dioxide; DGDG: Digalactosyldiacylglycerol; DGTS: Diacylglyceryltrimethylhomoserine; DNA: Deoxyribonucleic acid; ETC: Electron transport chain; FBA: Flux Balance Analysis; G6P: Glucose-6-phosphate; GAP: Glyceraldehyde-3-phosphate; GC-MS: Gas chromatography mass spectrometry; H^+^: Proton; HPLC: High performance liquid chromatography; KEGG: Kyoto Encyclopedia of Genes and Genomes; MGDG: Monogalactosyldiglycerol; NADH: Nicotinamide adenine dinucleotide; NADPH: Nicotinamide adenine dinucleotide phosphate; PE: Phosphoethanolamine; PG: Phosphatidylglycerol; PI: Phosphoinositol; PSI: Photosystem I; RNA: Ribonucleic Acid; Rubisco: Ribulose-1,5-bisphosphate carboxylase/oxygenase; SP: Signal Peptide; SQDG: Sulfoquinovosyldiacylglycerol; TCA: Tricarboxylic Acid

## Authors' contributions

NRB carried out all aspects of the work and drafted the manuscript. JAM conceived the study and participated in the design of the study and revisions of the manuscript. All authors read and approve the final manuscript.

## Supplementary Material

Additional file 1 **Metabolite list**. Comprehensive list of metabolites and abbreviations used in the metabolic reconstruction and FBA model for *C. reinhardtii*. Click here for file

Additional file 2 **Reaction list**. Comprehensive list of reactions used in the metabolic reconstruction and FBA model for *C. reinhardtii*. Click here for file

Additional file 3 **Amino acid compositions**. Amino acid compositions for each growth condition (auto-, hetero-, mixo-). Click here for file
